# Quantum Chemistry Based Simulation of Enantioseparation on Cyclodextrin‐ and Polysaccharide‐Based Chiral Stationary Phases

**DOI:** 10.1002/chem.202501398

**Published:** 2025-06-16

**Authors:** Linda Nelles‐Ziegler, Christoph Plett, Stefan Grimme

**Affiliations:** ^1^ Mulliken Center for Theoretical Chemistry, Clausius Institute for Physical and Theoretical Chemistry University of Bonn Beringstr. 4 53115 Bonn Germany

**Keywords:** chiral HPLC, computational chemistry, DFT, enantioseparation, molecular docking

## Abstract

We assess the capability of modern quantum chemical methods to simulate enantioseparation on chiral stationary phases (CSPs) in high‐performance liquid chromatography (HPLC) by comparing calculated and experimental elution orders (EEOs). Compared to previous studies, this work utilizes more accurate state‐of‐the‐art density functional theory (DFT) methods combined with automated computational workflows. The proposed approach employs molecular docking, conformer sampling, and DFT refinement for final ensemble‐based association free energy calculations of two diastereomeric complexes. Ten drug‐type molecules were considered on two common CSPs for which various molecular models were investigated. Although the association free energies of the strongest binding motifs were rather system‐dependen t ranging from about −9 to 29 kcal/mol, the differences between the two enantiomers were always only a few kcal/mol, sometimes even below 1 kcal/mol. Despite these small differences, correct determination of EEOs for all tested cyclodextrin‐based CSP systems was achieved. Even for more flexible polysaccharide‐based CSPs, the workflow yielded correct EEO results in 90% of the tested cases, provided that a sufficiently large cut‐out of the CSP material consisting of about 150 atoms was considered as a model. Due to the latter constraint, the method remains computationally expensive, requiring further research for improving practical application in, e.g., screening studies.

## Graphical abstract

We employed a systematic quantum chemistry‐based computational approach for simulating chiral HPLC, incorporating high‐level DFT methods. For the popular polysaccharide‐based CSPs, 90% of determined elution orders were computed correctly.

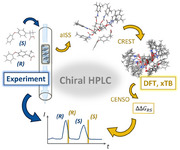

## 1 Introduction

Although enantiomers of a drug‐like compound share many physicochemical properties, their pharmaceutical effects can differ significantly.^[^ ^1^, ^2^, ^3^, ^4^ ^]^ Ensuring enantiomeric purity is therefore crucial for drug safety and efficacy, making chiral separation an integral step in their development and manufacturing.^[^ ^1^, ^2^, ^5^ ^]^

**Figure 1:**
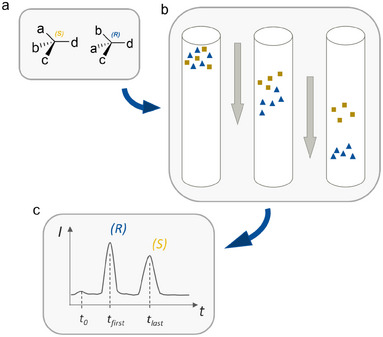
Concept of direct chiral HPLC. a) Enantiomers of drug‐like molecules, b) their separation on a chiral stationary phase, and c) resulting chromatogram with distinct elution times.^[^ ^6^ ^]^

A widely used technique for enantioseparation is direct chiral high‐performance liquid chromatography (HPLC), where an analyte mixture dissolved in a liquid mobile phase is forced under high pressure through a column containing a chiral stationary phase (CSP). The CSP's chiral properties result in different interactions with the enantiomers, leading to their separation and distinct elution times, which are plotted in a chromatogram (Figure 1).^[^ ^2^, ^5^, ^6^ ^]^

A measure for the separation performance is the selectivity factor α, defined by the ratio of enantiomer elution times, each corrected for the mobile phase elution time $t_{0}$ (Equation 1). α allows for comparison across chromatographic systems, as it is rather independent of experimental conditions,^[^ ^6^ ^]^

(1) $$\alpha =\frac{t_{last}-t_{0}}{t_{first}-t_{0}}.$$

Although the underlying enantio‐recognition mechanisms are not yet fully understood, it is well established that transient diastereomeric complexes form under quasi‐equilibrium conditions between the analyte and the CSP, exhibiting distinct complexation free energies for different enantiomers.^[^ ^7^ ^]^

The enantiomer binding more strongly to the CSP (with a more negative complexation free energy) is expected to elute later than the enantiomer binding more weakly. The complexation free energy of the analyte molecule in the CSP depends on steric constraints and non‐covalent interactions (NCIs), of which hydrogen bonding, dipole–dipole interactions, London dispersion, and hydrophobic effects have particular relevance.^[^ ^6^, ^8^, ^9^, ^10^ ^]^ These interactions do not occur solely between the analyte and the CSP but are also influenced by the mobile phase, and variations in its composition can therefore significantly impact selectivity.^[^ ^8^, ^10^, ^11^, ^12^ ^]^ This principle is exploited in HPLC method development, which relies heavily on empirical screening. Additional parameters that influence enantiomeric elution order (EEO) include the immobilization technique of the chiral selector, the direction from which the eluent composition is approached, and temperature effects.^[^ ^13^, ^14^, ^15^ ^]^

Although very time‐consuming, the screening process remains inevitable for each individual analyte, as reliably predicting separation performance for a specific chromatographic setup is not feasible yet.^[^ ^4^, ^5^, ^6^, ^9^, ^16^, ^17^, ^18^, ^19^ ^]^To address this issue, several theoretical studies over the past decades have focused on elucidating enantio‐recognition mechanisms, using various methods ranging from mechanistic simulations based on molecular mechanics or molecular docking to rather data‐driven approaches employing quantitative structure–enantioselective retention relationship (QSERR) models and machine learning algorithms.^[^ ^9^, ^16^, ^20^, ^21^, ^22^, ^23^, ^24^ ^]^Some of those studies have advanced the mechanistic understanding of enantioseparation, particularly in polysaccharide‐based systems.^[^ ^25^, ^26^, ^27^, ^28^, ^29^, ^30^, ^31^ ^]^ However, computation of chiral HPLC enantioseparation remains challenging due to several factors. First, the experiment is sensitive to pressure, temperature, and solvent variation, which requires accounting for molecular flexibility and solvation effects in the calculations. Second, the need for accuracy of the employed methods is high, as the separation process is often associated with very small differences in complexation free energy. Lastly, the process's dynamic, multi‐step nature, which involves numerous successive adsorption and desorption equilibrium events between analyte and CSP has to be addressed. ^[^ ^8^, ^16^, ^17^ ^]^

Considering these points, both most commonly used approaches, namely, classical molecular dynamics (MD) and molecular docking simulations^[^ ^9^, ^12^, ^17^, ^20^ ^]^, have inherent limitations: MD simulations are better suited to capture the iterative adsorption–desorption dynamics that characterize chiral HPLC; however, they lack the electronic, quantum chemistry‐based resolution needed for accurate interaction energy evaluation. On the other hand, accurate quantum chemical methods are most suitable for use in static molecular docking approaches using equilibrium structures.^[^ ^8^, ^16^, ^32^, ^33^ ^]^

In this study, we employed a quantum chemical approach that aims to enhance the accuracy compared to existing molecular docking studies, which were mostly limited to computationally rather cheap methods with comparably low accuracy such as force‐field‐based MD or low‐level density functional theory (DFT) and/or had a rather limited scope in terms of the number of systems considered. Studies using low‐level DFT typically modeled only small representations of polysaccharide‐based CSP materials. Further, we aim to improve the representation of dynamic behavior and entropic effects, as our study employs metadynamics‐based ensemble averaging over many different interacting structures and is therefore not purely static. However, the general shortcomings of a molecular docking method regarding larger‐scale dynamics, including the solvent molecules as occurring within a multi‐step chromatographic process, remain noteworthy.

Additionally, this study aimed for a more systematic approach regarding both the number of systems investigated and, in the case of the polysaccharide‐based CSPs, a comprehensive evaluation of different system sizes. The latter primarily aimed to minimize the computational cost, but ultimately also provided further insights into the relevant molecular interactions in enantioseparation.

### 1.1 Chiral Stationary Phases

**Figure 2:**
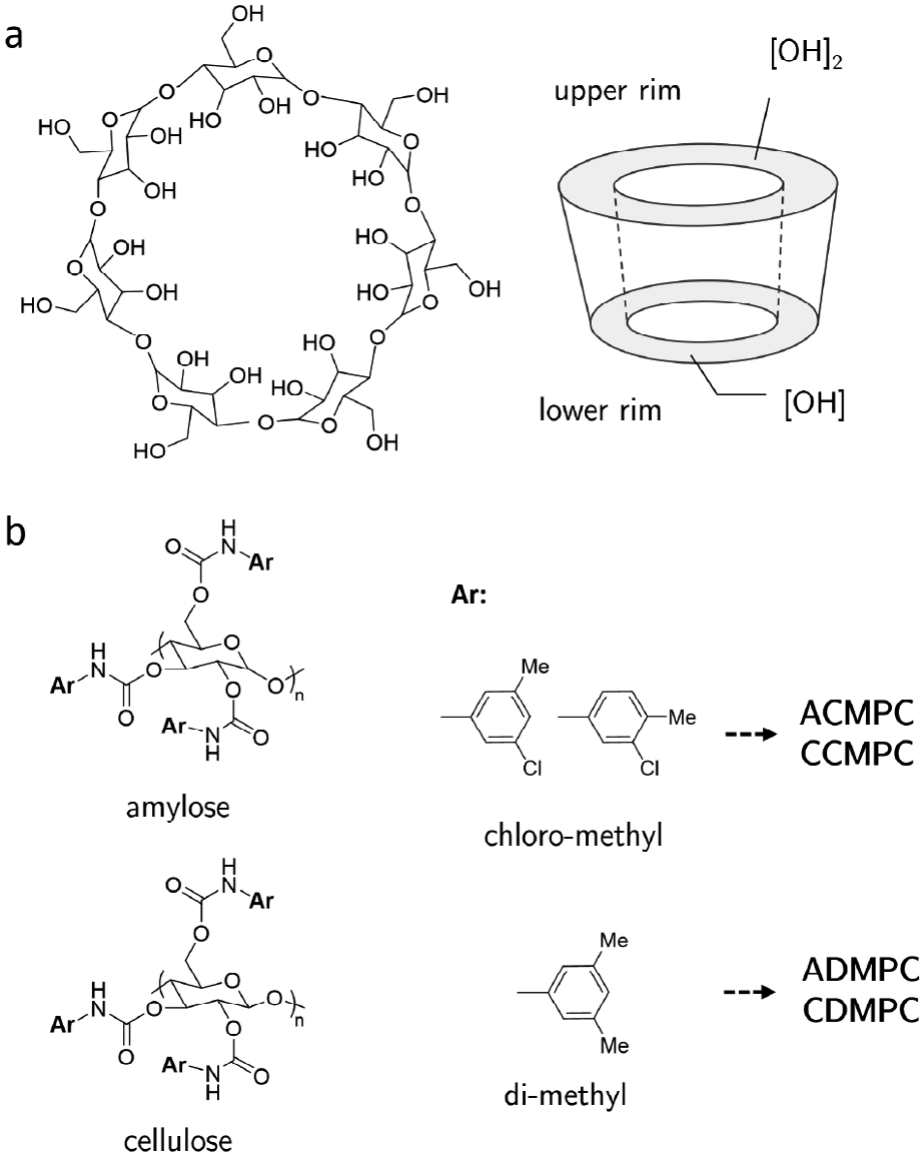
Structures of a) β‐CD and b) polysaccharide‐based CSP materials, including different backbone types and arylcarbamate selectors.

This study focused on cyclodextrin‐ and polysaccharide‐based CSPs. Cyclodextrins (CDs) are glucopyranose macrocycles that typically form inclusion complexes with analytes and can also engage in hydrogen bonding via ester groups on their rims.^[^ ^10^, ^34^ ^]^ A popular form of CD is a variant called β‐CD, which includes seven glucopyranose units (Figure 2). Although other macrocycle sizes and derivatized variants are also used in enantioseparation, we focused on native β‐CD, as its cavity size is suitable for a broad range of drug‐like analytes.^[^ ^34^ ^]^ Further, derivatized CDs are larger and more flexible, which substantially increases the computational cost.

Polysaccharide‐based CSPs typically consist of an amylose or cellulose backbone derivatized with arylcarbamates that create multi‐chiral cavities capable of adsorbing analyte molecules (Figure 2).^[^ ^4^, ^8^, ^9^, ^10^ ^]^ The carbamate selectors provide sites for hydrogen bonding, influenced by different types of phenyl substitution with chloro‐ and/or methyl‐groups, while the phenyl groups generally enable π–π stacking interactions.^[^ ^5^, ^9^, ^10^ ^]^ Variation of backbone and phenyl group substitution lead to entirely distinct separation behaviors, which is why commercial columns are available in a range of their combinations.^[^ ^6^ ^]^

A distinctive feature of polysaccharide‐based CSPs is that their higher‐order structure undergoes fundamental structural transitions with temperature and mobile phase variations, making these CSPs highly adaptable for the aforementioned screening procedures.^[^ ^8^, ^35^, ^36^ ^]^ This versatility makes this type of CSP the most commonly used in practice^[^ ^1^, ^8^, ^10^ ^]^, thus it was the main focus of this study.

### 1.2 Analytes

**Figure 3:**
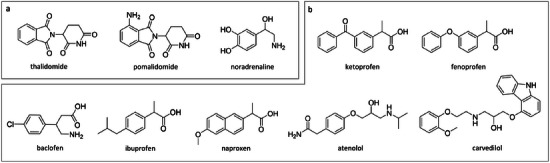
Investigated drug‐like analyte molecules with a) cyclodextrin‐based CSPs and b) polysaccharide‐based CSPs.

A total of 10 drug‐like molecules were investigated (Figure 3), selected based on the availability of relevant reference data. Three different systems with non‐derivatized β‐CD and confirmed 1:1 stoichiometry^[^ ^37^, ^38^, ^39^ ^]^ as well as 10 systems, including polysaccharide‐based CSPs were simulated, the latter covering both amylose‐ and cellulose‐based CSPs. For the polysaccharide‐based selectors, we specifically selected studies that included multiple chromatographic systems under comparable conditions, aiming to reduce heterogeneity in the experimental dataset. Although complete consistency across literature data cannot be ensured, this selection strategy was intended to minimize variability and improve the reliability of computed–experimental comparisons, particularly in the context of reported standard deviations.

Various cutout sizes were tested as CSP model, including a backbone trimer, a single carbamate selector including one glucose unit, a monomer of one glucose unit with three carbamate selectors, and a dimer consisting of two glucose units with a total of six carbamate selectors (for cutout structures, see Supporting Information, Figure S1). Selectors with different substitutions (3,5‐dimethyl in ADMPC and CDMPC, 3‐chloro‐4‐methyl in CCMPC, 3‐chloro‐5‐methyl in ACMPC) were considered (Figure S2, Supporting Information).

Most experimental studies used solvent mixtures, while in this work the solvent in the largest proportion (either $H_{2}O$, hexane, or MeOH) was modeled as many solvent models, like SMD, are not parameterized for mixtures. As in previous theoretical studies, the solid silica gel support was not considered in order to minimize computational cost.

## 2 Theoretical Methods

The applied workflow aims to determine the difference in binding free energies of two enantiomers to the CSP ($\Delta \Delta G_{RS}$) to derive an enantiomeric elution order (EEO). The binding free energies of the enantiomers ($\Delta G_{R}$, and $\Delta G_{S}$) were calculated from the absolute free energies of the enantiomer ($G_{guest})$, the CSP model ($G_{host})$, and their complexes ($G_{complex})$

(2) $$\Delta G_{R/S}=G_{complex}-\left(G_{guest}+G_{host}\right).$$

The free energies include contributions of electronic energy ($E_{\text{el}}$), free solvation energy ($\delta G_{\text{solv}}$), and thermostatistics according to the modified RRHO (rigid rotor harmonic oscillator) approximation ($\delta G_{\text{RRHO}}$) ^[^ ^40^, ^41^ ^]^

(3) $$G=E_{\text{el}}+\delta G_{\text{solv}}+\delta G_{\text{RRHO}}.$$

**Figure 4:**
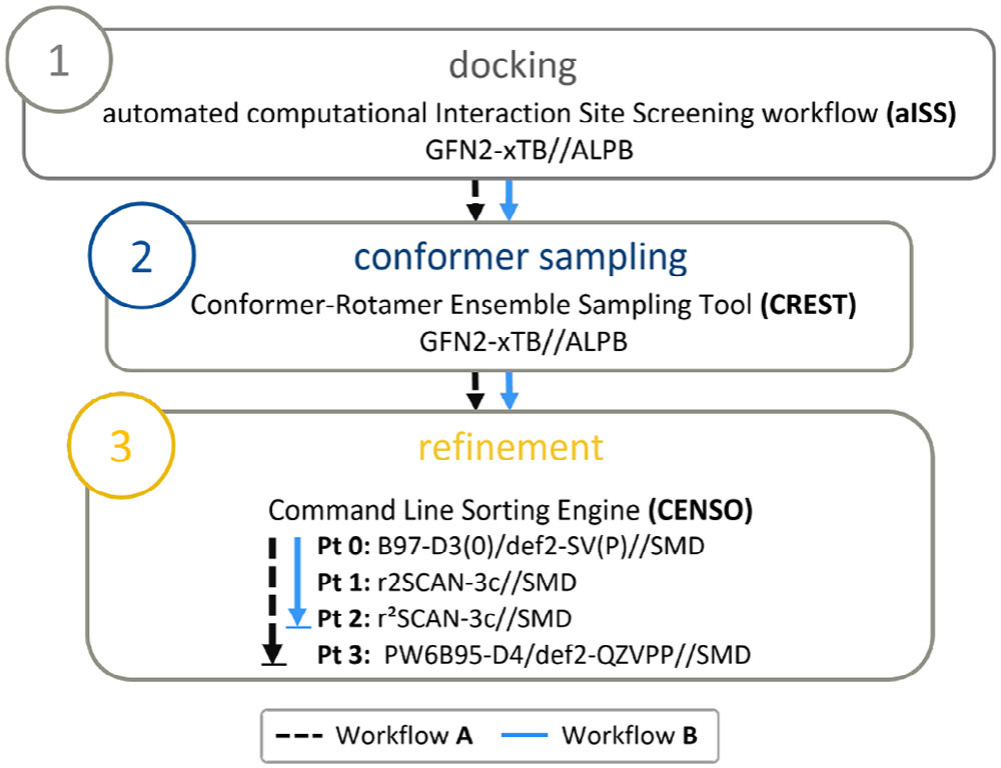
Multi‐step workflow used in this work for computing free energies with abbreviations of DFT computational levels given in parts 0–3 of the CENSO algorithm.

To generate the required molecular structures, a multi‐step workflow^[^ ^40^ ^]^ combining semi‐empirical and DFT methods (Figure 4) was employed ensuring that solvation effects were consistently accounted for by continuum models at all stages. It has been applied successfully in various computational studies of partition coefficients, pKa values, optical rotation, or calculation of NMR spectra.^[^ ^42^, ^43^, ^44^, ^45^ ^]^

Initial docking of the analyte to the CSP model employed the aISS (automated computational Interaction Site Screening) workflow with GFN2‐xTB optimizations and the ALPB solvation model.^[^ ^48^ ^]^ The conformational space of the analyte‐CSP complex was searched using CREST employing the GFN2‐xTB/ALPB method for the (meta)dynamics‐based sampling, and geometry optimization.^[^ ^32^, ^40^, ^49^ ^]^ CREST's NCI mode was applied in 50% of the runs to enhance the modeling of weak NCIs.^[^ ^50^ ^]^

Ensemble refinement was performed with the CENSO algorithm,^[^ ^40^ ^]^ employing DFT‐level optimization with $r^{2}\mathrm{SCAN}$‐3c^[^ ^51^ ^]^ and SMD solvation.^[^ ^52^ ^]^ Final electronic energies were determined using the hybrid DFT PW6B95^[^ ^53^ ^]^‐D4^[^ ^54^ ^]^/def2‐QZVPP^[^ ^55^ ^]^ in workflow A and the faster composite $r^{2}\mathrm{SCAN}$‐3c method in workflow B used for DFT geometry optimization, evaluating the necessity of refining the final single‐point energies with a computationally more costly hybrid functional. All systems were considered in their neutral state, thereby neglecting any effects of (de)protonation under the experimental conditions, an assumption that appears reasonable for the weakly acidic or basic drug‐like molecules studied here.

To reduce statistical uncertainties, two consecutive CREST and CENSO runs were performed for each enantiomeric complex in the case of the β‐CD CSPs, while four runs were conducted for the polysaccharide‐based CSP systems. The lowest final Boltzmann‐weighted free energies (at $T_{ref}$) from these runs were used as the final result for each complex. The average final ensembles consisted of 39 (workflow B) and 33 conformers (workflow A), respectively, while the average CREST ensemble contained 375 conformers (see Supporting Information Table S5).

As reference, experimental EEOs from literature were used. The calculated EEO was determined by identifying the enantiomer in the complex with the higher (less negative) complexation free energy as the first to elute as it binds less strongly to the CSP.

Further, selectivity factors α ^[^ ^8^, ^16^, ^17^ ^]^ were derived from the difference in complexation energies ($\Delta \Delta G_{RS}$) with the more stable complex referred to as last

(4) $$\Delta \Delta G_{RS}=\Delta G_{last}-\Delta G_{first}.$$

For this purpose, linear regression using a van't Hoff approach (Equation 5)^[^ ^5^, ^8^, ^35^, ^38^, ^56^ ^]^ was employed

(5) $$\Delta \Delta G_{RS}=-RTln\left(\alpha \right)$$

where R is the universal gas constant and T the absolute temperature. The determined values for α were compared to experimental values from the literature.

## 3 Results and Discussion

### 3.1 Cyclodextrins

All calculated complexation free energies for the β‐CD systems were negative (Table 1), indicating favorable analyte‐CSP complexation. In all cases, $\Delta G_{R}$ was greater than $\Delta G_{S}$, reflecting an EEO of *(R)* before *(S)*, consistent with experimental data. The EEOs were determined correctly with both workflows and only small differences in ΔG and ΔΔG values, respectively.

**Table 1:** Results for β‐CD systems. Complexation free energies $\Delta G_{R;S}$ and selectivities $\Delta \Delta G_{RS}$ in kcal/mol. Workflow A includes PW6B95‐D4/def2‐QZVPP//SMD single‐point energy refinement, workflow B contains $r^{2}\mathrm{SCAN}$‐3c//SMD single‐point energies after geometry optimization.

Analyte	Solvent	T/K	Workflow	$\Delta G_{R}$	$\Delta G_{S}$	$\Delta \Delta G_{RS}$	first	${\mathrm{first}}_{exp}$	α	$\alpha_{exp}$
Thalidomide	$H_{2}O$	278.15	A	−7.52	−8.73	1.21	R	R^[^ ^38^ ^]^	1.04	1.20^[^ ^38^ ^]^
			B	−8.58	−9.89	1.31	R		1.04	
Noradrenaline	$H_{2}O$	298.15	A	−3.38	−5.92	2.53	R	R^[^ ^46^, ^47^ ^]^	1.28	1.04^[^ ^47^ ^]^
			B	−5.46	−7.01	1.55	R		1.16	
Pomalidomide	$H_{2}O$	288.15	A	−7.56	−7.91	0.34	R	R^[^ ^39^ ^]^	1.03	1.12^[^ ^39^ ^]^
			B	−8.71	−9.20	0.49	R		1.05	

Workflow B consistently yielded lower complexation free energies than workflow A, reflecting a tendency of $r^{2}\mathrm{SCAN}$‐3c to yield slightly stronger NCIs than PW6B95‐D4/def2‐QZVPP for these systems.^[^ ^51^ ^]^

Comparing the NCI and normal runtype of CREST, using the NCI mode was necessary to yield correct EEOs. The energy differences between the lowest‐energy conformers identified in different CREST runs showed a maximum spread of 1.14 kcal/mol and a SD of 0.54 kcal/mol. However, such small deviations can become critical when examining the small free energy differences between two enantiomeric CSP complexes.

While the qualitative EEOs were found correctly, the agreement of experimental and theoretical selectivities α is only mediocre. The values were either overestimated (noradrenaline) or underestimated (thalidomide, pomalidomide). Since selectivity factors are rather sensitive to small differences in free energy, even minor residual errors in the calculations can lead to significant deviations from experimentally determined values. The remaining errors include, for example, insufficiently sized CSP cutouts or the neglect of the silica gel support in order to maintain computational feasibility.

All analytes formed the expected inclusion complexes with β‐CD. To verify the calculated structures, the analyte orientations within the cavity were compared to NMR data. For thalidomide and pomalidomide, the calculated orientations with the phenyl group facing the lower rim match those from the experiment.^[^ ^38^, ^39^ ^]^

**Figure 5:**
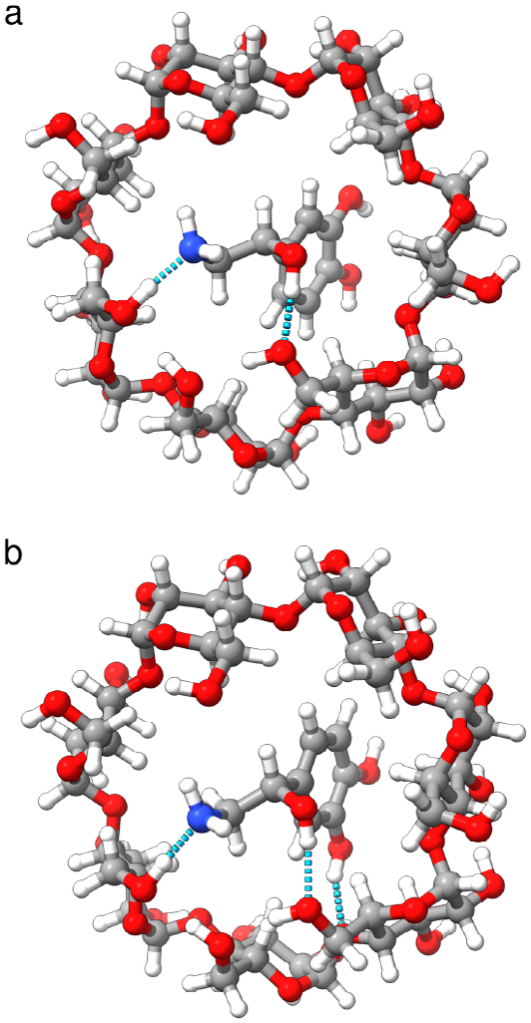
Optimized structures of β‐CD and *(R)*‐noradrenaline showing a) two (workflow A) and b) three hydrogen bonds as indicated by the dashed lines (workflow B).

Both workflows found the *(S)‐*noradrenaline with the phenyl group and *(R)*‐noradrenaline with the amino group facing the lower rim (Figure 5), consistent with the coexistence of both orientations in aqueous solution.^[^ ^37^ ^]^ In most cases, workflow B refined complexes with more hydrogen bonds than workflow A, highlighting some methodological differences in handling NCIs.

### 3.2 Polysaccharide‐Based CSPs

**Figure 6:**
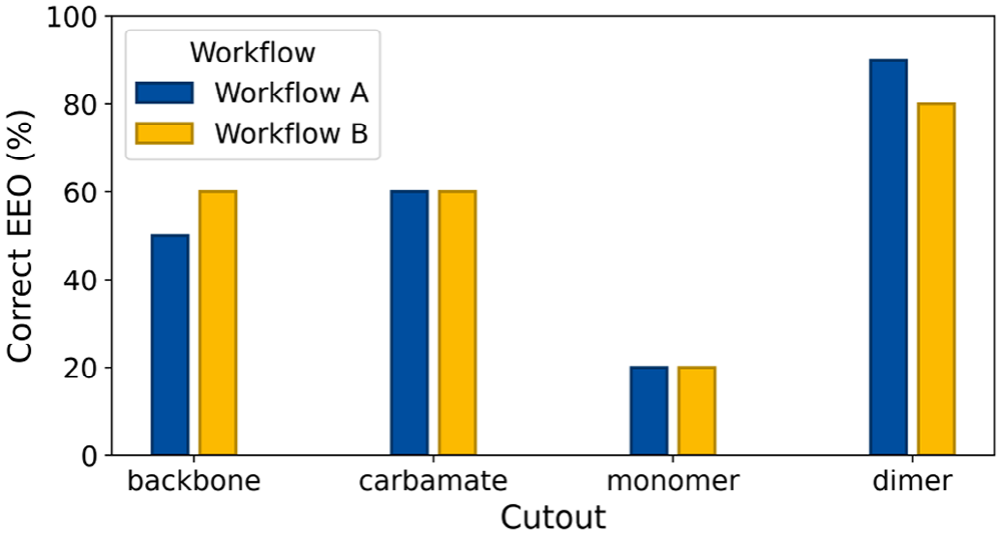
Percentage of correctly determined EEOs per polysaccharide‐based CSP cutout.

Figure 6 shows the percentage of correctly determined EEOs for polysaccharide‐based CSP systems across different cutouts. The dimer cutout enabled correct EEO determination in 90% (workflow A) and 80% (workflow B) of the tested cases. Accuracy was lowest with monomer cutouts for both workflows, while backbone and carbamate cutouts yielded 50%–60% accuracy. This shows that the dimer cutouts are essential for reaching high accuracy, likely due to their ability to form cavities. Further, at least the dimer cutout is required to capture both relevant backbone and selector variations. The results remained overall consistent between workflows, aligning with β‐CD system findings.

**Figure 7:**
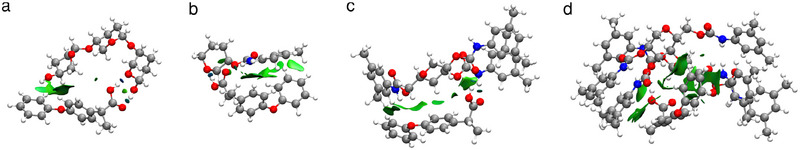
NCI plot analysis^[^ ^61^ ^]^ for typical equilibrium structures of CDMPC and fenoprofen complexes with the a) *(R)*‐enantiomer and backbone, b) *(R)*‐enantiomer and carbamate, c) *(S)*‐enantiomer and monomer and d) *(R)*‐enantiomer and dimer cutout. The color code is based on $sign(\lambda_{2})\rho$ and ranges from blue (‐0.7) for strong attractive interactions to green (0.0) for van der Waals interactions to red (0.7) for repulsive interactions. The density was plotted for an isovalue of 0.3 a.u.

Structures of representative complexes including an analysis of the non‐covalent interactions are shown in Figure 7. Generally, the NCIs are dominated by van der Waals interactions (green surface), but also strong attractive interactions are seen in all cutouts (blue surface) in the regions of hydrogen bonding. In case of the backbone cutout, the overall NCI contact is rather small. It increases for the carbamate and monomer cutout, yet no cavities were formed. In the dimer cutout, selectors stacked on top of each other, stabilizing the analyte and forming cavity‐like structures with hydrogen bonding and π--π‐stacking. These findings align with the EEO accuracy trends and the current understanding of steric and functional fit. Dimer complexes containing ACMPC showed cleavage sites in several cases after DFT optimization. For these three systems, significantly higher ΔG results were obtained in comparison (see Table 2).

**Table 2:** Results for polysaccharide‐based CSPs. Complexation free energies, EEOs, and selectivities for **dimer** cutouts. All energies are given in kcal/mol. Workflow A includes PW6B95‐D4/def2‐QZVPP//SMD single‐point energy refinement while workflow B contains the $r^{2}\mathrm{SCAN}$‐3c//SMD single‐point energies after geometry optimization.

Analyte	CSP	Solvent	T/K	WF	$\Delta G_{R}$	$\Delta G_{S}$	$\Delta \Delta G_{RS}$	first	${\mathrm{first}}_{exp}$	α	$\alpha_{exp}$
ibuprofen	CCMPC	hexane	298.15	A	0.31	−0.55	0.87	R	R^[^ ^35^ ^]^	1.09	1.06^[^ ^35^ ^]^
				B	−0.91	−1.68	0.77	R		1.08	
ibuprofen	ACMPC	$H_{2}O$	295.15	A	28.55	26.75	1.81	R	R^[^ ^57^ ^]^	1.20	1.17^[^ ^57^ ^]^
				B	27.69	25.14	2.56	R		1.28	
ketoprofen	ADMPC	hexane	298.15	A	−3.87	−3.64	0.23	S	R^[^ ^35^ ^]^	1.02	1.24^[^ ^35^ ^]^
				B	−5.39	−5.44	0.05	R		1.005	
ketoprofen	ACMPC	$H_{2}O$	295.15	A	24.80	26.36	1.57	S	S^[^ ^57^ ^]^	1.16	1.16^[^ ^57^ ^]^
				B	23.42	24.59	1.17	S		1.12	
fenoprofen	ADMPC	hexane	298.15	A	−2.75	−3.16	0.41	R	R^[^ ^35^ ^]^	1.04	1.10^[^ ^35^ ^]^
				B	−3.83	−4.00	0.17	R		1.02	
fenoprofen	CDMPC	hexane	298.15	A	0.94	0.28	0.67	R	R^[^ ^35^ ^]^	1.07	1.08^[^ ^35^ ^]^
				B	−1.85	1.21	0.64	S		1.06	
baclofen	ADMPC	$H_{2}O$	298.15	A	3.79	4.14	0.35	S	S^[^ ^58^ ^]^	1.03	n/a
				B	4.23	3.62	0.62	R		1.06	
naproxen	ACMPC	$H_{2}O$	295.15	A	27.66	25.15	2.50	R	R^[^ ^57^ ^]^	1.27	1.15^[^ ^57^ ^]^
				B	24.85	24.81	0.032	R		1.00	
atenolol	CDMPC	hexane	298.15	A	2.93	−1.47	4.40	R	R^[^ ^59^ ^]^	1.53	1.26^[^ ^59^ ^]^
				B	0.24	−3.44	3.68	R		1.43	
carvedilol	ADMPC	MeOH	298.15	A	5.14	4.22	0.92	R	R^[^ ^60^ ^]^	1.09	n/a
				B	3.24	1.82	1.42	R		1.15	

Table 2 shows the calculated complexation free energies ($\Delta G_{R;S}$) from the dimer cutouts. The differences in complexation energies ($\Delta \Delta G_{RS}$) are very small, which further highlights the importance of methodological accuracy and additionally numerical precision. As noted before, the free energies computed using workflow B are systematically lower than those obtained with workflow A.

To further analyze residual errors, a quantitative evaluation of the calculated selectivity factors was conducted for the systems with comprehensive reference data (Table 2). A detailed plot covering the differences between calculated and experimental selectivity factors regarding all cutouts is shown in the Supporting Information (Figure S3). Again, the quantitative analysis revealed a mixed picture, with both under‐ and overestimated selectivities. A potential source of error lies in the simplification of the system to a pure solvent model.

The average energy spread and conformational variability of various structural cutouts used in the CSP models were analyzed (Supporting Information, Table S4). Dimers exhibited the highest flexibility, with the largest standard deviation among the lowest‐energy CREST conformers, while carbamates were the most rigid. These findings are consistent with the expected flexibility and size of the respective fragments and underline the importance of performing accurate conformational searches.

### 3.3 Computation Times

**Figure 8:**
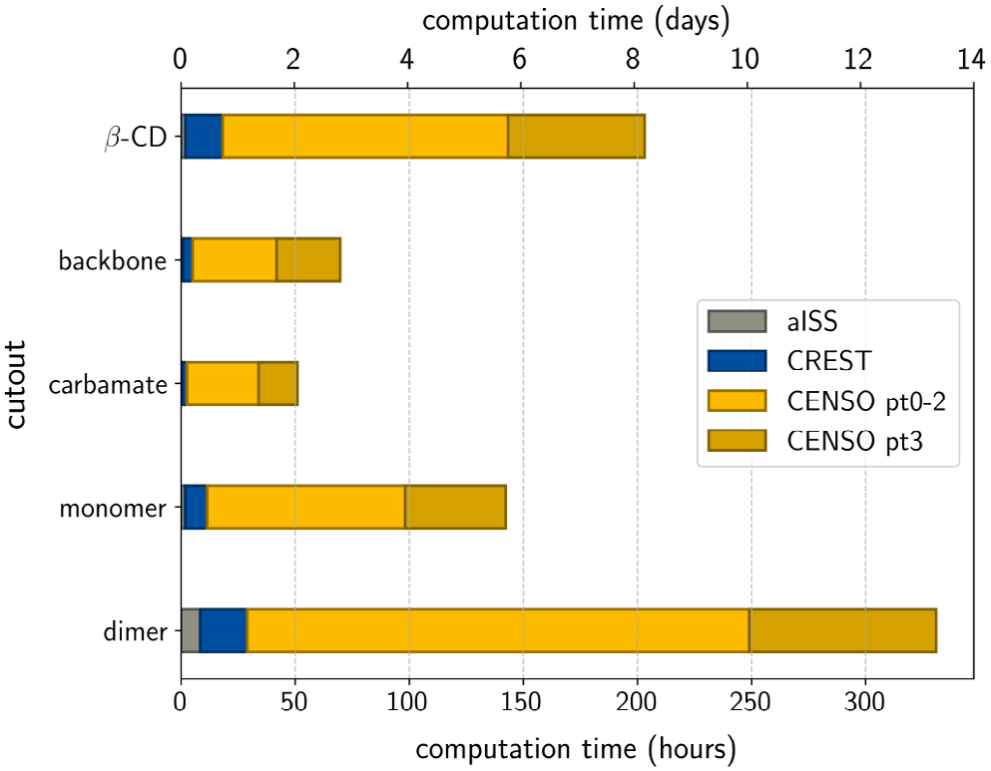
Huber mean computation times for β‐CD complexes and polysaccharide‐based CSP complexes across all cutouts.

The mean values of the computation times were calculated (Figure 8), based on all analyte‐CSP complex calculations using the Huber mean as defined in the Supporting Information.^[^ ^62^ ^]^

For β‐CD, workflow A totaled 203.2 hours (8.46 days). Skipping the refinement of energies using PW6B95‐D4/def2‐QZVPP//SMD in part 3 (workflow B) reduced computation by 30% to 143.3 hours (5.97 days) while still yielding a qualitatively correct EEO.

For the polysaccharide‐based CSP systems, the computational cost increased very strongly with the cutout size. In workflow A, total computation times were highest for the dimer (330.9 hours), followed by the monomer (142.3 hours), backbone (69.7 hours), and carbamate (51.03 hours). Workflow B significantly reduced these times: dimer (−24.7% to 249.0 hours), monomer (−31.0% to 98.2 hours), backbone (−39.9% to 41.9 hours), and carbamate (−33.4% to 34.0 hours).

## 4 Conclusion

We employed a systematic quantum chemistry‐based computational approach for determining EEOs and selectivities in chiral HPLC, using docking, conformer sampling, and free energy calculations with DFT refinement at different accuracy levels. The workflow was chosen to balance accuracy and computational cost by employing a multi‐step protocol that refines molecular interactions with implicit solvation and biased molecular dynamics sampling techniques.

The results demonstrate that the approach can reliably determine EEOs for cyclodextrin‐based chiral stationary phases (CSPs). For polysaccharide‐based CSPs, which dominate chiral HPLC applications, the method achieved up to 90% correct EEOs when using appropriate sized molecular cutouts as CSP model. Although quantifying selectivity remains challenging due to the subtle free energy differences governing enantioseparation, the workflow provides mechanistic insights into the role of non‐covalent interactions and steric constraints.

Our findings highlight that CSP cutouts of at least dimer size are necessary to capture the essential enantioselective effects, while smaller cutouts fail to provide sufficient steric complexity. This insight supports a more targeted selection of computational models for further studies. To our knowledge, this is the first systematic evaluation of fragment size effects in chiral HPLC modeling using state‐of‐the‐art quantum chemical methods combined with dynamic sampling. We acknowledge that using a pure solvent, omitting the silica support material, and modeling fragmented selectors are deliberate simplifications that limit the simulation of the full chromatographic process, especially its dynamic nature.

The choice of pure solvents was necessary due to the lack of parametrization for solvent mixtures in the employed solvation model (SMD), which may contribute to deviations between calculated and experimental selectivity values. The choice of fragments and neglect of support material was necessary to lower computational cost as much as possible. Nonetheless, the largely qualitatively correct results indicate that these approximations do not fundamentally compromise the validity of the conclusions. While the model does not simulate the full chromatographic process, it yields semi‐quantitative agreement with experimental data and helps define current strengths and limitations of QM‐based approaches to describe enantioseparation.

Even though computationally demanding, our approach represents an important step toward a computationally guided understanding of chiral separations. With ongoing advances in computational efficiency and theoretical modeling, automated applications can be realized, laying the foundation for developing a tool that seamlessly integrates into chiral HPLC method development.

## 5 Computational Details

The calculations were performed on 28 cores of an Intel Xeon E5‐2660 v4 (2.00 GHz).

The aISS workflow was used with xtb version 6.7.0. CREST was used in version 3.0.1 in combination with xtb version 6.7.0. CENSO was employed in version 1.2.0, using xtb version 6.7.0 and ORCA version 5.0.4.

Workflow B included CENSO parts 0 to 2, with final DFT‐level geometry optimization performed using $r^{2}\mathrm{SCAN}$‐3c//SMD and a filtering threshold of ΔG = 2.5 kcal/mol. Workflow A additionally included CENSO part 3, applying PW6B95‐D4/def2‐QZVPP//SMD and a 99.0% filtering threshold based on the Boltzmann distribution.

The NCI plot analysis was carried out with NCIPLOT version 4.2.^[^ ^63^ ^]^

If not stated otherwise, software defaults were applied.

## Conflict of Interest

The authors declare no conflict of interest.

## Supporting information

Supporting Information

## Data Availability

The data that support the findings of this study are available in the supplementary material of this article.
