# Supplementary material for: Quantum Chemistry Based Simulation of Enantioseparation on Cyclodextrin‐ and Polysaccharide‐Based Chiral Stationary Phases
Source: Chemistry. 2025 Jun 16;31(39):e202501398. doi: 10.1002/chem.202501398 (PMC12258693; doi:10.1002/chem.202501398)
Supplement: Supplementary file 1 — Supporting Information [file CHEM-31-e202501398-s001.pdf]

Towards Routine Quantum Chemistry based Simulation of Enantioseparation on Cyclodextrin- and Polysaccharide-based Chiral Stationary Phases

Linda Nelles-Ziegler,[†,‡] Christoph Plett,[†,‡] and Stefan Grimme[*,†]

†*Mulliken Center for Theoretical Chemistry, 53115 Bonn, Germany*

‡*These authors contributed equally*

E-mail: grimme@thch.uni-bonn.de

S1 Statistical error measures

In this work, the following statistical measures were used.

Statistical measure for a set $x_1, \cdots, x_n$ of data points with references $r_1, \cdots, r_n$ are:

Mean deviation (MSD):

$$MD = \frac{1}{n}\sum_{i}^{n}(x_i - r_i) \tag{1}$$

Mean absolute deviation (MAD):

$$MAD = \frac{1}{n}\sum_{i}^{n}|x_i - r_i| \tag{2}$$

Standard deviation (SD):

$$SD = \sqrt{\frac{1}{n-1}\sum_{i=1}^{n}\left((x_i - r_i) - MD\right)^2} \tag{3}$$

Huber Mean ($L_\delta(y, \hat{y})$):

$$L_\delta(y, \hat{y}) = \begin{cases} 0.5(y - \hat{y})^2, & \text{for}|y - \hat{y}| \leq \delta \\ \delta\left(|y - \hat{y}| - 0.5\delta\right), & \text{otherwise.} \end{cases} \tag{4}$$

S2 Supporting Tables

Table S1: Polysaccharide-based CSPs - Complexation free energies and EEOs for backbone cutouts. All energies are given in kcal/mol. Workflow A (WF) includes PW6B95-D4/def2-QZVPP//SMD single-point energy refinement while workflow B contains the r^{2}SCAN-3c//SMD single-point energies after geometry optimization.

Analyte	CSP	Solvent	T/K	WF	ΔG_R	ΔG_S	$\Delta\Delta G_{RS}$	first	α
ibuprofen	CCMPC	hexane	298.15	A	0.98	2.31	1.33	S	1.14
				B	-0.46	1.22	1.68	S	1.18
ibuprofen	ACMPC	H_2O	295.15	A	3.49	1.62	1.88	R	1.20
				B	2.38	1.46	0.92	R	1.09
ketoprofen	ADMPC	hexane	298.15	A	0.45	0.16	0.29	R	1.03
				B	-1.84	-2.01	0.18	R	1.02
ketoprofen	ACMPC	H_2O	295.15	A	3.47	1.01	0.86	R	1.09
				B	1.46	0.55	0.91	R	1.09
fenoprofen	ADMPC	hexane	298.15	A	2.55	1.65	0.90	R	1.09
				B	0.54	-0.57	1.11	R	1.11
fenoprofen	CDMPC	hexane	298.15	A	-0.37	0.91	1.29	S	1.13
				B	-2.07	-0.19	1.88	S	1.20
baclofen	ADMPC	H_2O	298.15	A	10.58	4.71	5.87	R	1.76
				B	11.84	4.69	7.15	R	1.99
naproxen	ACMPC	H2O	295.15	A	2.26	3.02	0.76	S	1.08
				B	1.74	1.12	0.62	R	1.06
atenolol	CDMPC	hexane	298.15	A	0.95	-0.46	1.41	R	1.15
				B	-1.83	-1.88	0.055	R	1.005
carvedilol	ADMPC	MeOH	298.15	A	9.45	7.62	1.84	R	1.19
				B	7.47	5.20	2.27	R	1.24

Table S2: Polysaccharide-based CSPs - Complexation free energies and EEOs for carbamate cutouts. All energies are given in kcal/mol. Workflow A (WF) includes PW6B95-D4/def2-QZVPP//SMD single-point energy refinement while workflow B contains the r^{2}SCAN-3c//SMD single-point energies after geometry optimization.

Analyte	CSP	Solvent	T/K	WF	ΔG_R	ΔG_S	$\Delta\Delta G_{RS}$	first	α
ibuprofen	CCMPC	hexane	298.15	A	1.75	1.68	0.068	R	1.01
				B	.0058	.0059	.00013	R	1.00
ibuprofen	ACMPC	H_2O	295.15	A	1.74	2.37	0.64	S	1.06
				B	0.15	1.29	1.14	S	1.12
ketoprofen	ADMPC	hexane	298.15	A	1.72	1.66	0.055	R	1.005
				B	-0.17	0.17	0.35	S	1.03
ketoprofen	ACMPC	H_2O	295.15	A	1.70	1.53	0.17	R	1.02
				B	0.29	0.14	0.15	R	1.01
fenoprofen	ADMPC	hexane	298.15	A	2.29	1.72	0.56	R	1.06
				B	0.89	0.36	0.53	R	1.05
fenoprofen	CDMPC	hexane	298.15	A	2.29	1.72	0.56	R	1.06
				B	0.89	0.36	0.53	R	1.05
baclofen	ADMPC	H_2O	298.15	A	3.28	2.45	0.83	R	1.08
				B	3.71	2.01	1.70	R	1.18
naproxen	ACMPC	H_2O	295.15	A	-0.41	-0.71	0.30	R	1.03
				B	-0.23	-0.78	1.05	R	1.05
atenolol	CDMPC	hexane	298.15	A	2.05	0.86	1.18	R	1.12
				B	0.21	-2.07	2.28	R	1.25
carvedilol	ADMPC	MeOH	298.15	A	4.00	3.90	0.11	S	1.01
				B	3.35	-0.35	3.70	R	1.43

Table S3: Polysaccharide-based CSPs - Complexation free energies and EEOs for monomer cutouts. All energies are given in kcal/mol. Workflow (WF) A includes PW6B95-D4/def2-QZVPP//SMD single-point energy refinement while workflow B contains the r^{2}SCAN-3c//SMD single-point energies after geometry optimization.

Analyte	CSP	Solvent	T/K	WF	ΔG_R	ΔG_S	$\Delta\Delta G_{RS}$	first	α
ibuprofen	CCMPC	hexane	298.15	A	-1.16	-1.03	0.13	S	1.01
				B	-2.57	-1.99	0.58	S	1.06
ibuprofen	ACMPC	H_2O	295.15	A	-0.73	-0.97	0.23	R	1.03
				B	-1.02	-1.84	0.82	R	1.08
ketoprofen	ADMPC	hexane	298.15	A	-2.20	-1.86	0.35	S	1.03
				B	-4.52	-3.89	0.62	S	1.06
ketoprofen	ACMPC	H_2O	295.15	A	-0.24	-0.82	0.85	R	1.06
				B	-1.77	-2.60	0.84	R	1.08
fenoprofen	ADMPC	hexane	298.15	A	-1.88	-1.18	0.70	S	1.07
				B	-4.36	-2.71	1.64	S	1.17
fenoprofen	CDMPC	hexane	298.15	A	-1.88	-1.18	0.70	S	1.07
				B	-4.36	-2.71	1.64	S	1.17
baclofen	ADMPC	H_2O	298.15	A	1.20	-1.48	2.67	R	1.29
				B	0.087	-2.43	2.52	R	1.27
naproxen	ACMPC	H_2O	295.15	A	-1.94	0.68	2.62	S	1.29
				B	-2.12	-0.06	2.07	S	1.22
atenolol	CDMPC	hexane	298.15	A	-3.28	-0.72	2.56	S	1.28
				B	-6.39	-5.54	0.85	S	1.09
carvedilol	ADMPC	MeOH	298.15	A	4.10	2.53	1.57	R	1.16
				B	2.62	0.53	2.09	R	1.22

Table S4: Average energy spread ($\Delta E_{\text{max-min}}$) and standard deviation (SD) in kcal/mol of conformer energies (complexes) after the four CREST runs for different cutout types.

Cutout	$\Delta E_{\text{max-min}}$	SD
backbone	4.4113	1.6150
carbamate	1.3582	0.5634
monomer	2.0826	0.7109
dimer	9.3015	3.4154

Table S5: Average number of conformers after the different stages for various cutout types.

Cutout	CREST ensemble	workflow A	workflow B
dimer	304	16	12
backbone	496	51	45
carbamate	315	52	45
monomer	386	40	34
Total	375	39	33

S3 Supporting Figures

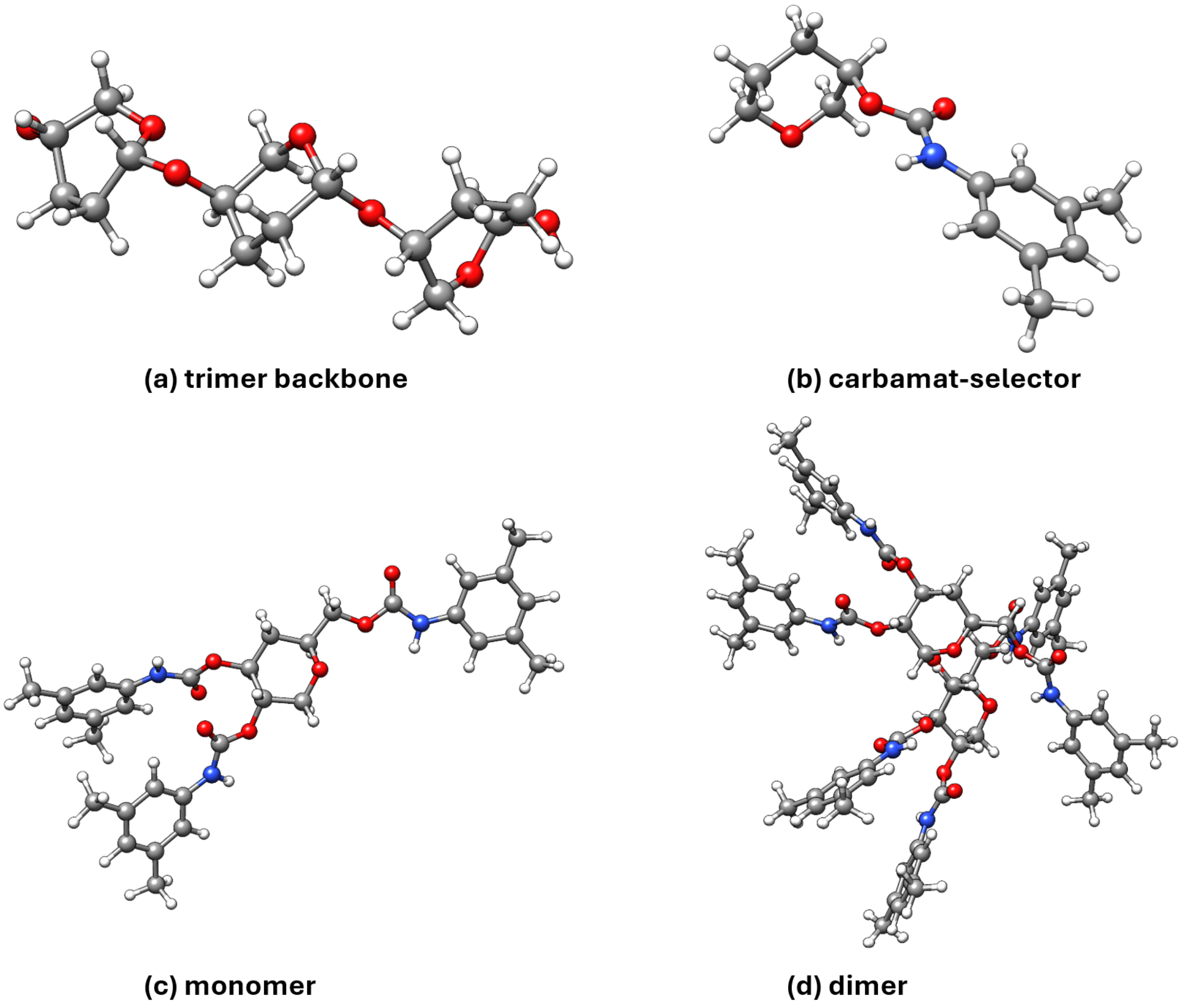

Figure S1: Investigated Cutout Types of Polysaccharide-based CSPs.

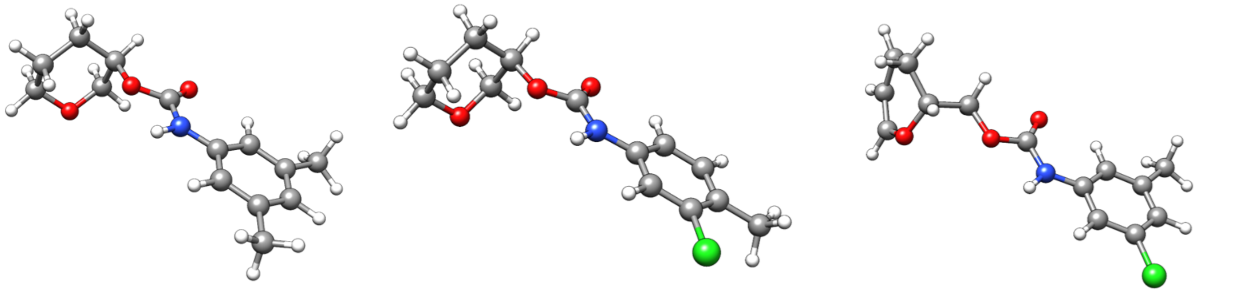

selector (a)
3,5-dimethylphenylcarbamate

selector (b)
3-chloro-4-methylphenylcarbamate

selector (c)
3-chloro-5-methylphenylcarbamate

Figure S2: Investigated Selector Types of Polysaccharide-based CSPs.

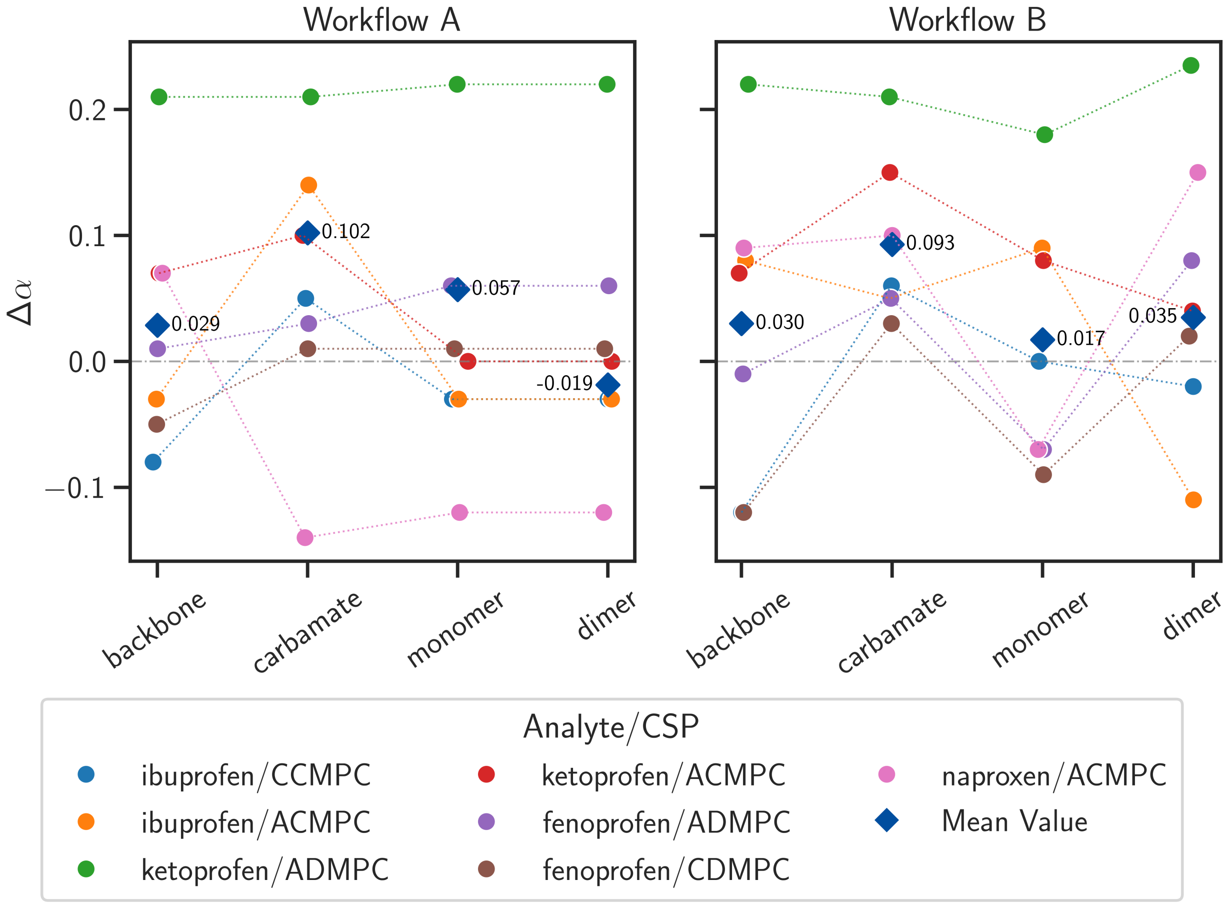

Figure S3: Absolute differences $\Delta\alpha = \alpha_{ref} - \alpha_{calc}$ regarding all cutouts, and both workflows.
